# Early impacts of the Pennsylvania Rural Health Model on potentially avoidable utilization

**DOI:** 10.1093/haschl/qxae002

**Published:** 2024-01-19

**Authors:** Donald S Bourne, Eric T Roberts, Lindsay M Sabik

**Affiliations:** Department of Health Policy and Management, University of Pittsburgh School of Public Health, Pittsburgh, PA 15261, United States; Division of General Internal Medicine, University of Pennsylvania Perelman School of Medicine, and Leonard Davis Institute of Health Economics, Philadelphia, PA 19104, United States; Department of Health Policy and Management, University of Pittsburgh School of Public Health, Pittsburgh, PA 15261, United States

**Keywords:** Pennsylvania Rural Health Model, global budgets, health policy, prevention quality indicators, difference-in-differences

## Abstract

The Pennsylvania Rural Health Model (PARHM) is a novel alternative payment model for rural hospitals that aims to test whether hospital-based global budgets, coupled with delivery transformation plans, improve the quality of health care and health outcomes in rural communities. Eighteen hospitals joined PARHM in 3 cohorts between 2019 and 2021. This study assessed PARHM’s impact on changes in potentially avoidable utilization (PAU)—a measure of admission rates policymakers explicitly targeted for improvement in PARHM. Using a difference-in-differences analysis and all-payer hospital discharge data for Pennsylvania hospitals from 2016 through 2022, we found no significant overall reduction in community-level PAU rates up to 4 years post–PARHM implementation, relative to changes in rural Pennsylvania communities whose hospitals did not join PARHM. However, heterogeneous treatment effects were observed across cohorts that joined PARHM in different years, and between critical access vs prospective payment system hospitals. These findings offer insight into how alternative payment models in rural health care settings may have heterogeneous impacts based on contextual factors and highlight the importance of accounting for these factors in proposed expansions of alternative payment models for rural health systems.

## Introduction

Nearly 1 in 5 Americans (∼60 million people) live in rural areas.^1^ Rural residents depend on local hospitals as an important, and sometimes the only, source of care in their communities.^2^ However, rural hospitals’ financial and operational challenges have increasingly contributed to suboptimal access to and quality of care.^3-5^ Given limited access to high-quality primary and outpatient specialty care, compared with their urban counterparts, people who live in rural communities are more likely to experience potentially avoidable utilization (PAU) for chronic conditions.^6,7^

The Pennsylvania Rural Health Model (PARHM) is the first alternative payment model (APM) designed specifically for hospitals serving rural populations. The PARHM seeks to test whether hospital global budgets—a model in which hospitals are paid a prospective budget to cover all hospital-based services—in conjunction with hospital-specific health care delivery transformation plans, can sustain access to care and improve the quality of health care in rural communities. This model was launched in 2019, in partnership between the Center for Medicare and Medicaid Innovation (CMMI) and the Commonwealth of Pennsylvania, and is scheduled to run through 2024.^8,9^

The PARHM was developed to preserve and improve access to care for rural residents while catalyzing changes in care delivery that improve population health outcomes. Conceptually, the adoption of a global budget incentivizes hospitals to effectively manage resource-intensive hospital care, such as preventing PAU through coordinated ambulatory care. Within the PARHM framework, Pennsylvania also established specific targets for reducing PAU, which were included as a quality measure in hospital performance evaluations.^8,9^ The model included an upfront budget adjustment to hospitals, which reflected spending-reduction targets associated with lowered PAU. Hospitals also were mandated to develop transformation plans outlining how they would address population health needs, many of which focused on reducing PAU through improved coordination and engagement efforts with community-based organizations, community health care providers, and social service providers.^8,9^

There is growing interest in expanding the use of global budgets for rural hospitals to provide more sustainable financing and direct resources towards improving population health.^10,11^ However, evidence on the impacts of global budget payment models for rural hospitals is limited as Maryland is the only other state to institute hospital global budgets. Evidence on changes in PAU rates associated with Maryland’s global budget program inconsistently showed reductions and most of these studies included all Maryland hospitals, rather than focusing on rural facilities.^12-15^ Further, Maryland has a small rural population, lacks critical-access hospitals (CAHs), and its global budgets cover 100% of patient revenue.^16^ In PARHM, there is no centralized hospital rate setting, and because payer participation is voluntary the global budgets do not cover 100% of patient revenue.^8,9^ These differences make PARHM more generalizable to rural areas across the United States where policymakers have considered establishing similar programs. Therefore, we examined the impact of PARHM on changes in PAU rates, using a difference-in-differences (DiD) design that accounted for the staggered entry of hospitals into the model.

## Policy context and conceptual framework

The PARHM is a global budget payment model in which participating hospitals receive predetermined (ie, prospective) payments from participating payers to cover hospital-based inpatient and select outpatient hospital services.^8,9^ (See Appendix 1 for a more detailed description of PARHM.)

Participation in PARHM is voluntary, and hospitals in Pennsylvania were eligible for PARHM if they were located in a county defined as rural by the Center for Rural Pennsylvania (ie, population density <284 persons per square mile).^8,9^ Each hospital’s global budget is the sum of global budget amounts from each participating payer as set by the Commonwealth and CMMI. Amounts are based on historical net patient revenue from each payer. For CAHs, final budgets were adjusted to reflect the hospital’s actual costs in the previous year.^8^ For prospective payment system (PPS) hospitals, global budgets were adjusted based on several factors, including unplanned changes in volume or payer mix, planned changes to service lines, and other adjustments.^8^ The PARHM set a revenue scale target, aiming to place at least 75% of hospital revenue under a global budget in 2019, and at least 90% from 2020 to 2024.^8,9^

Policymakers introduced PARHM with the expectation that it would provide hospitals with stable financing, incentivize reductions in PAU, and channel resources toward addressing community health needs. Specifically, the shift to global budgets provides a predictable revenue stream not tied to volume and removes a hospital’s incentives to admit patients in order to generate additional revenue, as is the case under fee-for-service.^17^ Combined with transformation plans, global budgets are intended to shift hospital incentives towards addressing population health needs (eg, tailor service lines and programs to their patient population and community health needs) and reducing PAU through improvements in ambulatory care. Conceptually, these incentives have the potential to create a positive cycle as savings under a global budget (eg, from reducing PAU) can be reinvested to preserve or enhance services that address population needs and result in future savings.

Our objective was to quantify the effects of PARHM on changes in PAU rates. Based on the model’s design, we hypothesized that hospital service areas (HSAs) with PARHM-participating hospitals would experience a reduction in rates of PAU compared to control HSAs with hospitals that are PARHM-eligible, but non-participating in the model.

## Data and methods

### Study design and data sources

We performed a retrospective analysis of changes in PAU rates across the HSAs of all PARHM eligible hospitals. We used the Callaway and Sant’anna^18^ DiD with multiple time periods approach. This analysis quantifies the impact of PARHM on changes in PAU across rural populations served by participating hospitals relative to changes in areas served by eligible non-participating hospitals, while accounting for the staggered entry of hospitals into the program. The University of Pittsburgh Institutional Review Board approved this study.

Our primary data source was visit-level inpatient discharge data from the Pennsylvania Health Care Cost Containment Council (PHC4) from January 1, 2016, through December 31, 2022. All licensed health care facilities in Pennsylvania are required to report administrative data to PHC4, which is validated by standard processes and verified by the reporting facility. PHC4 contains visit information, including diagnoses, which we used to identify PAU.

We included several additional data sources. First, we obtained hospital financial data from PHC4 Financial Analysis Reports.^19^ Second, we obtained hospital characteristics from PHC4 Facility datasets (eg, number of licensed beds). Third, we used American Community Survey (ACS) 5-year estimates from 2016 through 2021 to obtain area-level demographic and socioeconomic measures, which we linked to PHC4 data at the HSA level.^20^ We used ACS 5-year estimates because these provide greater statistical reliability for less-populated areas. Fourth, the Dartmouth Atlas was used to construct HSAs, which are collections of contiguous ZIP codes identifying areas where residents receive most care from a specific hospital, representing local markets for hospital care.^21^ (See Appendix 2 for a more detailed description of data sources.)

### Outcome variables

We measured PAU using the Agency for Healthcare Research and Quality Prevention Quality Indicators (PQIs),^22^ which are a widely used indicator for assessing PAU and conceptually reflect the adequacy of access to and quality of ambulatory care in a geographic area.^22-24^ The primary outcome was an overall composite measure for all PQIs reported as a rate (number of PAU visits per 100 000 residents) at the HSA-year level. Secondary outcomes included acute, chronic, and diabetes composite measures, which are subsets of the overall composite measure. These PQIs are a component of hospital transformation plans and quality-reporting metrics under PARHM.^8,9^ (See Appendix 3, Table S1, for a full description of PQI measures.)

### HSA and hospital variables

We included several variables to characterize the demographic and economic attributes of HSAs and hospitals. From the ACS, we included median household income and the percentage of residents who were non-White, married, uninsured, high school graduates, unemployed, and with incomes below the poverty level. We also used the ACS to calculate the Area Deprivation Index (ADI) and Community Assets and Relative Rurality Index (CARR). The ADI was included as a measure of socioeconomic disadvantage, with higher values representing greater levels of socioeconomic deprivation.^25^ The CARR was used as a continuous measure of rurality that accounts for community services and infrastructure, with higher values indicating greater remoteness and less availability of services.^26^

Finally, we included the following characteristics of hospitals within each HSA: participation in the 340B program (which entitles hospitals to purchase drugs at discounted rates), hospital reimbursement methodology (CAH, which uses cost-based reimbursement, or PPS, which uses a predetermined reimbursement per admission based on diagnosis-related groups for inpatient services), hospital total margin, uncompensated care share of net patient revenue (NPR), Medicare share of NPR, Medicaid share of NPR, and discharge rates. We selected these variables because they differed between hospitals that did vs did not join PARHM and may be related to trends in hospital volume and revenue. (See Appendix 2 for a more detailed description of variables)

### Statistical analyses

We used a DiD design where the unit of analysis was the HSA-year. This design compares changes in outcomes in the treated group (ie, HSAs with PARHM-participating hospitals) from before to after model entry with contemporaneous changes among the control group (ie, HSAs with PARHM-eligible but non-participating hospitals). The assumption is that changes in the control cohort are a reasonable estimate of changes that would have been expected in the treated cohort in the absence of global budgets (ie, a valid counterfactual). Importantly, DiD controls for time-invariant differences between treated and control groups, and for time-varying factors that equally affected the cohorts.

Because hospitals joined PARHM in different years, we used the Callaway and Sant’anna^18^ DiD approach. This approach is an extension of the standard DiD design, which compares changes in outcomes over time between a treatment group and a control group. The Callaway and Sant’anna DiD takes this a step further by estimating treatment effects within multiple periods and multiple groups and then aggregating these results into an overall DiD estimate. This overall estimate represents an average treatment effect of PARHM pooled across HSAs. The approach avoids biases that can arise from comparisons of units entering treatment at different times when effects vary across cohorts or over time.^27-29^

**Figure 1.**
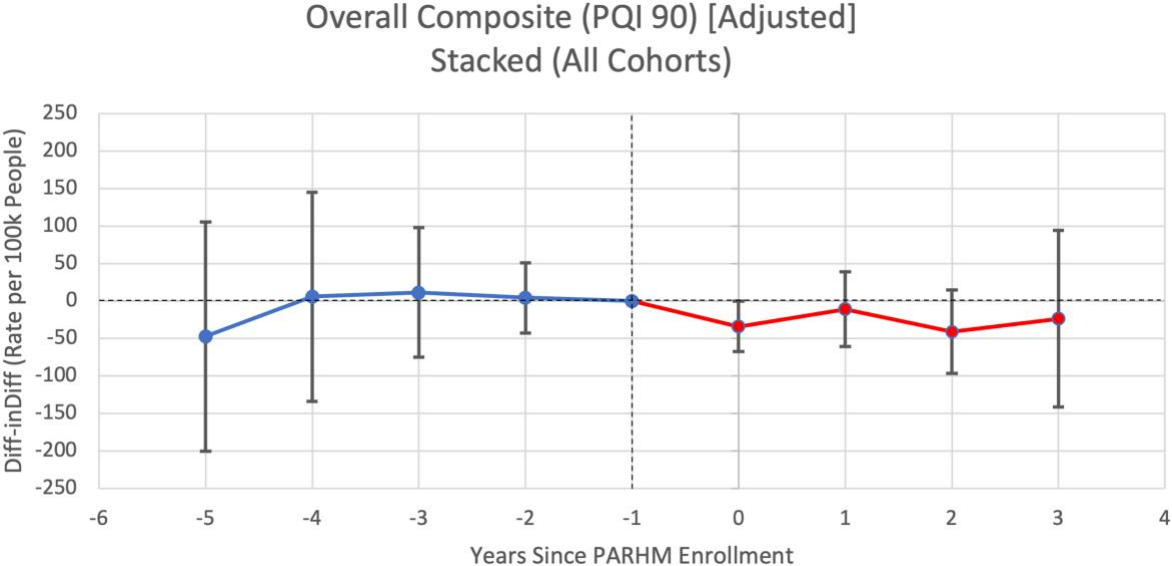
Pooled event study graph for prevention quality indicator overall composite rates per 100 000 people per hospital service area-year. Source: Authors’ analysis of Pennsylvania Health Care Cost Containment Council data (2016–2022). Point estimates are displayed along with their 95% CIs. Year 0 represents the first year of PARHM enrollment (ie, 2019 for cohort 1, 2020 for cohort 2, 2021 for cohort 3). Reference periods are the year before PARHM enrollment, set to zero, represented at year −1. Blue represents the pretreatment period and red represents the post-treatment period. Abbreviations: Diff-inDiff, difference-in-differences; PARHM, Pennsylvania Rural Health Model; PQI, Prevention Quality Indicator.

We estimated weighted models using a composite of population weights and propensity score weights to estimate the average treatment effect on the treated (ATT)—that is, a treatment effect among HSAs whose hospitals participated in PARHM. Population weights account for differences in population size across HSAs. We used propensity score methods to construct inverse probability weights to weight control HSAs to resemble treated HSAs on demographic, socioeconomic, and hospital characteristics in the pre-intervention period (see Appendix 4, Table S1, and Figure 1).^30^ Standard errors were clustered by HSA to account for arbitrary serial correlation.^31^

We used an event-study model to analyze dynamic outcome trends and test for trend differences between treatment and control groups in the pre-intervention period. Common, or “parallel,” pre-intervention trends suggest that the treatment and control groups likely would have continued to follow similar trajectories in the post-period without global budgets. (See Appendix 4 for a more detailed discussion of our statistical approach.)

Sensitivity analyses were conducted to test the robustness of our findings and included the following: (1) specifying an alternate start date 1 year prior to the implementation of PARHM to evaluate potential treatment anticipation; (2) removing 2020–2021 data to account for changes in care patterns due to the COVID-19 pandemic, including a statewide moratorium on elective surgeries,^32^ federal policies to stabilize hospital finances in 2020, and the Delta/Omicron surges in 2021; (3) using alternative estimation methods, such as ordinary least squares, inverse probability weighting, inverse probability weighting with stabilized weights, not yet treated HSAs as controls (ie, including HSAs from cohorts 2 and 3 as controls for cohort 1, and using HSAs from cohort 3 as controls for cohort 2); and (4) estimating unadjusted models by removing ATT propensity score weighting and covariates (other than fixed effects for HSAs and years).

Subgroup analyses were conducted to examine potential heterogeneity in treatment effects and included (1) restricting the sample to HSAs that meet a stricter definition of rural as used by the Federal Office of Rural Health Policy (FORHP)^33^ and (2) stratifying the analysis between CAHs and PPS hospitals to account for differences in global budget methodology.^8,9^

### Limitations

Our study has several limitations. First, selection bias is a potential concern because participation in PARHM was voluntary for eligible hospitals, and previous research identified baseline financial differences between participating and non-participating hospitals.^34,35^ We used propensity score weighting to account for factors (eg, HSA demographics and hospital finances) that could be related to model participation and pretreatment trends.^36^ Second, COVID-19 may have differentially affected the treatment and control HSAs after implementation of PARHM. We conducted a sensitivity analysis by removing data from the years 2020–2022 and the results were not appreciably different. Third, hospitals during our study period received federal financial assistance under the Coronavirus Aid, Relief, and Economic Security Act. These funds were intended to stabilize hospital finances by providing hospitals with prospective payments based on historical revenues, acting in a similar fashion to the global budgets in PARHM. Because hospitals in all HSAs had access to these relief funds, this may have attenuated differences in effective exposure to global budgets—and thus biased our estimates—towards the null. Finally, our study may not be generalizable to the national rural population or rural hospitals in other states. However, population demographics and rural hospitals in Pennsylvania are similar to those of rural communities elsewhere in the country.^37^

## Results

### Descriptive statistics

Table 1 summarizes the characteristics of HSAs in the study sample. We included all HSAs with PARHM-eligible hospitals between 2016 and 2022: 18 treated HSAs (ie, with PARHM-participating hospitals) and 45 control HSAs (with non-participating PARHM-eligible hospitals). Across treated and control HSAs, the analysis sample consisted of 441 HSA-year observations.

**Table 1.** Comparison of hospital service area demographics and characteristics at baseline (2016–2018).

	PARHM HSAs (*n* = 18)	Control HSAs (*n* = 45)	*P*
Demographics			
Total population, thousands	32.14	52.26	<.001^a^
Median income, $ in thousands	61.13	60.79	.743
High school graduate, %	89.79	88.11	.119
Unemployed, %	6.43	6.19	.360
Non-White, %	3.84	3.75	.846
Uninsured, %	12.80	13.15	.623
Below poverty level, %	10.90	11.19	.459
Married, %	53.86	53.96	.881
Characteristics			
ADI*^b^*	0.59	0.52	.221
CARR^b^	0.59	0.42	.190
FORHP rural	0.78	0.47	<.001^a^
Critical access hospital, *n* (%)	5 (27.78%)	10 (22.22%)	.421
Independent hospital,^c^*n* (%)	11 (61.11%)	7 (15.56%)	<.001^a^
340B participant, *n* (%)	9 (50.00%)	15 (33.33%)	.028^a^
Inpatient discharge rate, per *100 000*	1975.52	2509.58	.197
Outpatient discharge rate, per 100 000	4461.54	6699.34	.175
Total margin,^d^ %	−0.02	0.03	.001^a^
Uncompensated care share of NPR,^e^ %	2.67	2.40	.174
Medicare share of NPR,^f^ %	45.57	41.76	.006^a^
Medicaid share of NPR,^g^ %	13.22	12.68	.694

Abbreviations: ADI, Area Deprivation Index; CARR, Community Assets and Relative Rurality Index; FORHP, Federal Office of Rural Health Policy; HSA, hospital service area; NPR, net patient revenue; PARHM, Pennsylvania Rural Health Model.

Source: Authors’ analysis of American Community Survey and Pennsylvania Health Care Cost Containment Council data (2016–2018).

^a^Significant at the *P* < .05 level, 2-sample *t* test.

^b^ *z*-scored.

^c^Independent (ie, not merged or affiliated with another hospital or system).

^d^The ratio of total income to total revenue.

^e^Percentage of uncompensated care (charity care and bad debt) relative to net patient revenue.

^f^Percentage of Medicare revenue relative to net patient revenue.

^g^Percentage of Medicaid revenue relative to net patient revenue.

Apart from population size, population demographic characteristics were similar between the treated and control HSAs at baseline (Table 1). Compared with control HSAs, hospitals in treated HSAs were more likely to be independent (ie, not part of a system), have negative total margins, participate in the 340B drug pricing program, and have a greater Medicare share of net patient revenue. These baseline differences narrowed with propensity score weighting (Appendix 4, Table S1.) Both treated and control HSAs had similar PAU rates prior to PARHM (584.07 vs 582.36 PAU rates per 100 000 people per HSA-year; *P* = .96). (See Appendix 5, Table S1.)

### Primary outcome

**Figure 2.**
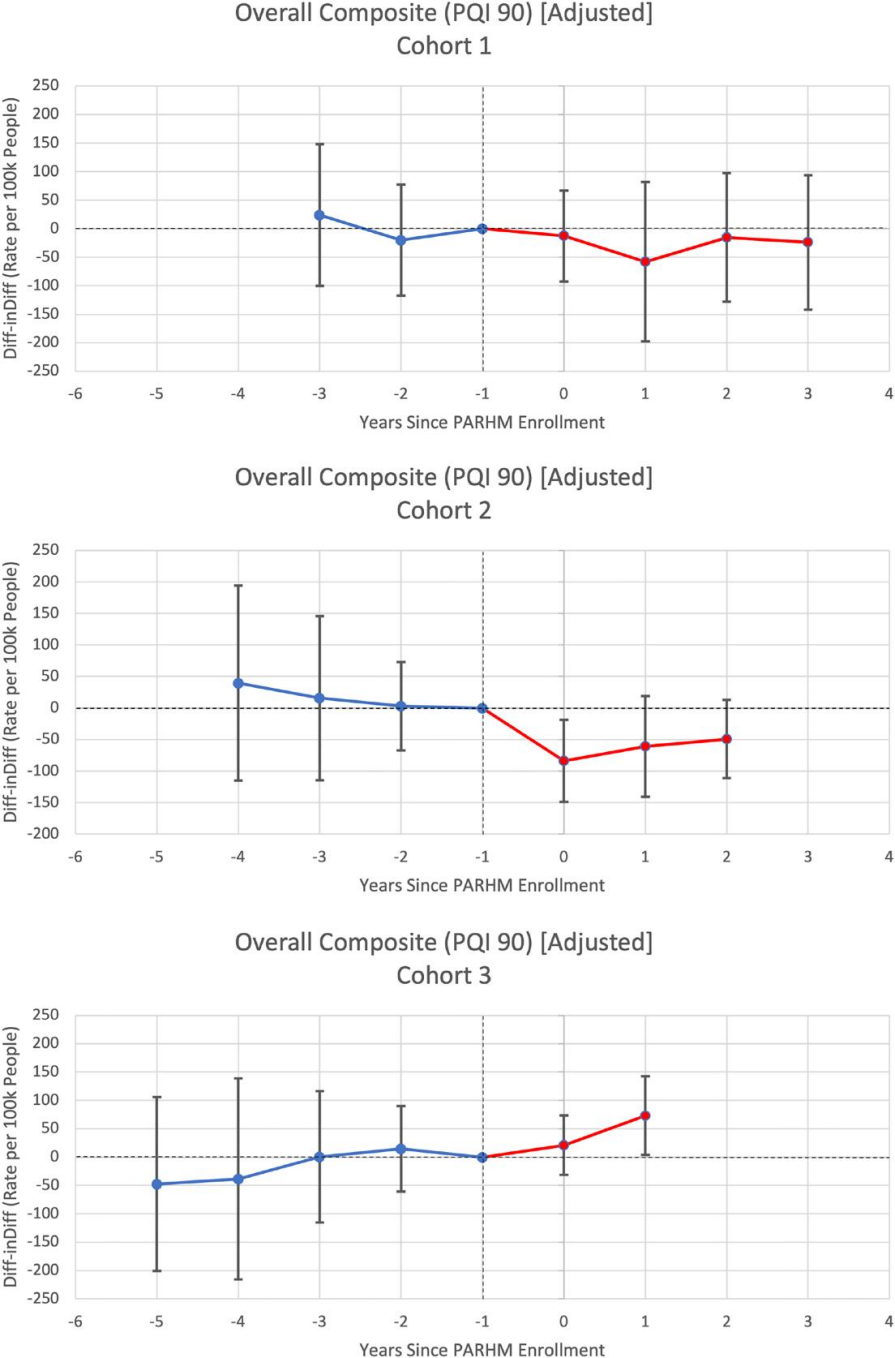
Individual cohort event study graphs for prevention quality indicator overall composite rates per 100 000 people per hospital service area-year. Source: Authors’ analysis of Pennsylvania Health Care Cost Containment Council data (2016–2022). Point estimates are displayed along with their 95% CIs. Year 0 represents the first year of PARHM enrollment (ie, 2019 for cohort 1, 2020 for cohort 2, 2021 for cohort 3). Reference periods are the year before PARHM enrollment, set to zero, represented at year −1. Blue represents the pretreatment period and red represents the post-treatment period. Abbreviations: Diff-inDiff, difference-in-differences; PARHM, Pennsylvania Rural Health Model; PQI, Prevention Quality Indicator.

Figure 1 plots estimates from an event study model that compared changes in overall PAU rates (ie, composite measure) between the treated and control HSAs from before to after hospitals entered PARHM (pooled across cohorts). Table 2 stratifies DiD estimates by cohort and Figure 2 plots estimates from an event study model for cohort-specific analyses. There was no evidence of differential pre-trends (ie, differential changes in PAU rates between treated and control HSAs prior to PARHM participation) and statistical tests revealed no violation of the parallel pre-trends assumption. Pooled across cohorts and post-treatment periods, there was a nonsignificant differential change in the PAU rate between treated and control HSAs from the pre- to the post-treatment periods (DiD estimate: −26.99 PAU per 100 000 people per HSA-year; 95% CI, −65.35 to 11.37) (Table 2).

**Table 2.** Difference-in-differences estimates (potential avoidable utilization per 100 000 people per hospital service area-year) pooled and across individual cohorts.

Potentially avoidable utilization	PARHM cohort 1 ATT (95% CI)	PARHM cohort 2 ATT (95% CI)	PARHM cohort 3 ATT (95% CI)	Pooled ATT (95% CI)
Overall composite admission rates	−27.38 (−116.19 to 61.42)	−64.86^a^ (−126.83 to −2.88)	46.99 (−11.57 to 20.97)	−26.99 (−65.35 to 11.37)
Acute composite admission rates	−8.28 (−45.72 to 29.16)	−20.09 (−41.08 to 0.91)	6.54 (−7.90 to 20.97)	−10.44 (−22.98 to 2.09)
Chronic composite admission rates	−19.10 (−77.35 to 39.15)	−44.77 (−97.20 to 7.77)	40.45 (−12.74 to 93.65)	−16.55 (−48.75 to 15.65)
Diabetes composite admission rates	−6.21 (−25.41 to 13.00)	−0.31 (−11.44 to 10.83)	12.82^a^ (4.06 to 21.57)	1.88 (−7.24 to 11.01)

Abbreviations: ATT, average treatment effect on the treated; PARHM, Pennsylvania Rural Health Model; PQI, Prevention Quality Indicator.

Source: Authors’ analysis of Pennsylvania Health Care Cost Containment Council data (2016–2022). “Pooled” reports the aggregated measure of treatment effects across all cohorts. See Appendix 5, Table S1, for baseline PAU rates.

^a^Significant at the *P* < .05 level.

Cohort 1 HSAs exhibited a small and nonsignificant differential decrease in PAU compared with control HSAs (Table 2). Cohort 2 HSAs experienced a statistically significant differential decrease of −64.86 PAU per 100 000 residents per HSA-year (95% CI, −126.83 to −2.88), compared with control HSAs. Cohort 3 HSAs followed a nonsignificant upward trend in PAU rates.

### Secondary outcomes

Table 2 lists adjusted DiD estimates for acute, chronic, and diabetes composite PAU measures. Event study graphs from Appendix 6, Figures S1–S3, show that baseline trends in PAU rates were not significantly different from zero. In the pooled analyses, there are insignificant differential changes in PAU rates during the pre- and post-treatment periods. Among the individual cohort analyses, only cohort 3 HSAs experienced a statistically significant differential increase in PAU for the Diabetes Composite (PQI 93) measure, 12.82 per 100 000 people per HSA-year (95% CI, 4.06 to 21.57) or 17.13%, compared with control HSAs. Cohort 3 HSAs generally followed a nonsignificant upward trend in PAU rates.

### Sensitivity analyses

Appendix 7, Table S1, shows a full list of results from our sensitivity analyses for our primary outcome, Overall Composite (PQI 90) measure. We found no significant differential change in overall PAU rates between treated and control HSAs, pooled or between cohorts, when testing for treatment anticipation, removal of 2020 data, and unadjusted models. When using alternative estimation methods, we continued to find a differential decrease in PAU rates in cohort 2 HSAs and a differential increase in cohort 3 HSAs compared with control HSAs.

### Subgroup analyses

Appendix 7, Table S2, shows a full list of results from our subgroup analyses for our primary outcome, Overall Composite (PQI 90) measure. We found no significant differential change in PAU rates between treated and control HSAs when limiting our sample to HSAs meeting the FORHP definition of rural. Among HSAs with PPS hospitals, results showed a significant differential decrease in PAU rates among cohort 2 HSAs, and among HSAs with CAHs, results showed a significant differential decrease in PAU rates among cohort 1 HSAs.

A concern in this analysis is that the effects of PARHM may be difficult to distinguish from the effects of the COVID-19 pandemic, particularly in 2020 and early 2021. To examine this issue, we obtained hospital-level admission data for confirmed COVID-19 cases from the Centers for Medicare and Medicaid Services and tested for differences in admission rates between treated and control HSAs, and across cohorts. No significant differences in COVID-19 hospitalization rates were observed (Appendix 8, Tables S1 and S2.)

## Discussion

This study of rural hospitals participating in PARHM, a hospital global budget model, found no overall differential reduction in PAU rates over a period of 2–4 years following the model’s implementation, relative to changes in a within-state control group. However, we observed heterogeneous treatment effects across cohorts. Specifically, in HSAs served by the second cohort of hospitals, we found a statistically significant differential decrease in overall PAU rates relative to control HSAs, while there was a nonsignificant differential increase in PAU in the third cohort relative to control HSAs. These heterogeneous findings were replicated across alternative specifications. Subgroup analyses identified heterogeneity in treatment effects between CAH and PPS hospitals. These findings suggest that PARHM was associated with limited changes in PAU rates overall while highlighting considerable heterogeneity in effects.

The lack of significant overall findings might be explained, in part, by factors such as the limited penetration of global budgets and PARHM’s payment methodology, under which hospitals faced potential adjustments to their budgets over time. Based on data from 2019, only 60% of participating hospitals met the revenue scale target (ie, that a hospital’s global budget accounted for at least 75% of eligible net patient revenue).^9^ This meant that hospitals were still impacted by volume changes after the implementation of PARHM. For CAHs, there was a significant lag (in some cases, >2 years) in determining final budget adjustments, which was reported to have impacted these hospitals’ ability to implement transformation plans.^9^ The proportion of CAHs by cohort ranged from 0% to 60%, which may have contributed to the heterogeneous results between cohorts.^8^ Further, because hospitals’ budgets could be adjusted to reflect volume changes (and for CAHs, cost changes), hospitals faced some uncertainty in the prospect of retaining long-run savings from reducing utilization or costs, which could have impacted performance under the model.

Other factors that differed between cohorts, such as hospital management, financial performance prior to PARHM, years of participation in PARHM, system affiliation, and other characteristics, may have further contributed to heterogeneous findings. Previous work reported that cohort 2 hospitals were larger and in a stronger financial position than cohort 1 hospitals before they joined PARHM.^35^ This could have enabled cohort 2 hospitals to adjust to the model and make investments to implement care transformation plans, including resources towards reducing PAU. However, additional research is needed to understand whether and how such factors contributed to differences in hospital performance under PARHM.

Notably, the effects of PARHM did not appear to increase within our study period. External constraints, such as inadequate access to primary care and vital outpatient specialty care, may not be directly addressed by PARHM, which may limit the model’s impact on PAU. It can take time for hospitals to adjust to a new payment model and implement transformation plans, such as hiring staff and improving access to outpatient services to reduce PAU.^8,9,38^ The PARHM is still maturing and hospitals that joined the model later may need further time to implement changes in care delivery. Trends from 2020 onwards might be obscured by factors related to COVID-19, which disrupted model implementation—especially in the later cohorts that joined the program during COVID-19. Additional work is needed to see if there is a sustained trend over the entire model demonstration period, which is scheduled to run through December 2024.

Findings from this study add to prior research on hospital global budget models. For example, evaluations of Maryland’s hospital global budget program found inconsistent evidence of reductions in hospital utilization. One study found a 5% reduction in admissions among Medicare patients over the model’s first 3 years and a 4% reduction among commercially insured population over 2 years relative to a comparison group.^16^ A second study with a 4-year follow-up period also found reductions in PAU rates in the Medicare population, but no difference among those who were commercially insured.^39^ Meanwhile, studies focusing on rural hospitals reported no differential changes in acute hospital use.^14,40,41^

Contextual factors, such as PARHM being specifically designed for rural hospitals, the incorporation of a hospital transformation plan, variation in treatment timing, the demographics of rural Pennsylvania, or other unmeasured factors, may account for the different results between Pennsylvania and Maryland. From a methodological perspective, the variation in rollout (staggered phase-in across rural areas) and a within-state cohort of PARHM-eligible hospitals (a natural control group) allowed us to control for state-level trends, which evaluators of Maryland’s program (a statewide model) could not control for. It is also important to note that PARHM is a complex and evolving program.^8,9,42^ For example, in response to the COVID-19 pandemic, CMMI granted flexibility in the model for budget adjustments and decreased the number of quality metrics required to be reported to reduce administrative burdens on hospitals.^9^

Our results provide the first evidence of PARHM’s impact on population health outcomes. This research is an important first step in understanding the health impacts of PARHM in its early years and highlights important considerations for policymakers contemplating similar models, such as the recently announced States Advancing All-Payer Health Equity Approaches and Development (AHEAD) Model from CMMI. States should take into consideration the compositions of their population and hospitals, model eligibility criteria, and the length of time it may take to see measurable results.

Future research should assess additional factors related to model impacts, including details regarding model implementation, transformation plans, health system affiliation, and other hospital and contextual factors that contribute to the model’s impacts. Assessments should examine the consistency of these findings as the program matures and other patient-centered health outcomes, such as indicators of inpatient quality and patient safety. Finally, it will be imperative to assess PARHM’s potential effect on rural–urban disparities in health care.

## Conclusion

The introduction of global budgets in rural Pennsylvania hospitals was not associated with statistically significant relative changes in PAU rates overall during the early years of program implementation. However, we did find evidence of treatment effect heterogeneity across groups of hospitals entering the model in different years and between CAHs and PPS hospitals. Continued evaluation of hospital performance under this payment model, and an understanding of the policy and contextual factors that may contribute to heterogeneity in program effects, can help inform the evolution of APMs for rural health care organizations.

## Supplementary Material

qxae002_Supplementary_Data
