# Supplementary material for: Early impacts of the Pennsylvania Rural Health Model on potentially avoidable utilization
Source: Health Aff Sch. 2024 Jan 19;2(2):qxae002. doi: 10.1093/haschl/qxae002 (PMC10836154; doi:10.1093/haschl/qxae002)
Supplement: qxae002_Supplementary_Data [file qxae002_Supplementary_Data.zip › Appendix 1.8.24 CLEAN.docx]

**Appendix to Early Impacts of the Pennsylvania Rural Health Model on Potentially Avoidable Utilization**

Table of Contents

[Appendix 1: Background on the Pennsylvania Rural Health Model 1](#_Toc155311483)

[Goals of PARHM 1](#_Toc155311484)

[Model Details 1](#_Toc155311485)

[Eligibility 1](#_Toc155311486)

[Timeline 1](#_Toc155311487)

[Oversight 2](#_Toc155311488)

[Components 2](#_Toc155311489)

[Potentially Avoidable Utilization Incentives 4](#_Toc155311490)

[Changes in Response to COVID-19 4](#_Toc155311491)

[PARHM Participants 4](#_Toc155311492)

[Appendix 1 Figure 1. Map of Participating Hospitals 5](#_Toc155311493)

[Appendix 1 Table 1. List of Participating Hospitals 6](#_Toc155311494)

[Participation Goals 6](#_Toc155311495)

[Appendix 2: Detailed description of data sources 7](#_Toc155311496)

[Appendix 3: Agency for Healthcare Research and Quality Prevention Quality Indicators 8](#_Toc155311497)

[Appendix 3 Table 1. Measures of Potentially Avoidable Utilization 8](#_Toc155311498)

[Appendix 4: Detailed overview of our statistical approach 9](#_Toc155311499)

[Appendix 4 Table 1. Standardized Mean Differences of Covariates with Average Treatment Effect on the Treated and Propensity Score Weighting 10](#_Toc155311500)

[Appendix 4 Figure 1. Distribution of Propensity Scores 11](#_Toc155311501)

[Appendix 5: Baseline Period Data 12](#_Toc155311502)

[Appendix 5 Table 1. Pre- and Post-Treatment Potentially Avoidable Hospitalization Rates per 100,000 people per Hospital Service Area-Year 12](#_Toc155311503)

[Appendix 6: Secondary Analyses 13](#_Toc155311504)

[Appendix 6 Figure 1. Event Study Graph for Prevention Quality Indicator Acute Composite Rates per 100,000 people per Hospital Service Area-Year 13](#_Toc155311505)

[Appendix 6 Figure 2. Event Study Graph for Prevention Quality Indicator Chronic Composite Rates per 100,000 people per Hospital Service Area-Year 14](#_Toc155311506)

[Appendix 6 Figure 3. Event Study Graph for Prevention Quality Indicator Diabetes Composite Rates per 100,000 people per Hospital Service Area-Year 15](#_Toc155311507)

[Appendix 7: Sensitivity and Subgroup Analyses 16](#_Toc155311508)

[Appendix 7 Table 1. Sensitivity Analyses: Difference-in-Differences Estimates (Potentially Avoidable Hospitalization Rates per 100,000 people per Hospital Service Area-Year) Pooled and Across Individual Cohorts 16](#_Toc155311509)

[Appendix 7 Table 2. Subgroup Analyses: Difference-in-Differences Estimates (Potentially Avoidable Hospitalization Rates per 100,000 people per Hospital Service Area-Year) Pooled and Across Individual Cohorts 17](#_Toc155311510)

[Appendix 8: COVID-19 Data 18](#_Toc155311511)

[Appendix 8 Table 1. Total Confirmed COVID-19 Admissions 18](#_Toc155311512)

[Appendix 8 Table 2. Confirmed COVID-19 Admission Rate per 100,000 people per Hospital Service Area-Year 19](#_Toc155311513)

[References 20](#_Toc155311514)

# Appendix 1: Background on the Pennsylvania Rural Health Model

Below we summarize key details of the Pennsylvania Rural Health Model (PARHM) to provide additional context for the analysis and findings presented in “Early Impacts of the Pennsylvania Rural Health Model on Potentially Avoidable Utilization.” These details were drawn primarily from PARHM evaluation reports from NORC at the University of Chicago, which provide further detail on program background, structure, and impacts.^1-3^

## Goals of PARHM

The model seeks to test whether care delivery transformation in conjunction with hospital global budgets can achieve the following goals^1-3^:

1. Increase Access to High-Quality Care: The model aims to enhance rural Pennsylvanians' access to high-quality care and improve their health.
   1. Increase access to primary and specialty care^4^
2. Improve the Financial Viability of Rural Hospitals: The model seeks to constrain the growth of hospital expenditures across payers, while stabilizing hospital finances to facilitate investments in care aligned with community health needs.
   1. Achieve $35 million in cumulative Medicare hospital savings over the course of the model^4^
   2. Limit hospital cost of care growth on inpatient and outpatient hospital-based services per beneficiary to no more than 3.38% annualy^4^
3. Improve Population Health Outcomes and Quality Targets: Pennsylvania commits to achieving targets related to population health outcomes and quality of care, such as reducing rural health disparities through improved chronic disease management and preventive screenings.
   1. Reduce rural health disparities through improved chronic disease management and preventive screenings^4^
   2. Reduce potentially avoidable utilization^3^
   3. Decrease deaths from substance use disorder and improve access to treatment for opioid abuse^4^

## Model Details

### Eligibility

All critical access hospitals (CAHs) and acute care hospitals paid under the inpatient and outpatient prospective payment system (PPS) located in a rural county, as defined by the Center for Rural Pennsylvania (i.e., 284 persons per square mile as of the 2010 Census), were eligible for PARHM.^1^ CAHs are small rural hospitals that receive cost-based reimbursement from Medicare, whereas PPS hospitals receive payment rates that are prospectively set according to the type and severity of an admission. Overall, our analysis included 65 eligible hospitals in 63 hospital service areas.

### Timeline

The model was announced in 2017. It was slated to run for six years between January 1, 2019 and December 31, 2024.

### Oversight

The Centers for Medicare & Medicaid Services Innovation (CMMI) oversees and monitors the implementation of PARHM. To help hospitals plan for and implement the model, the federal government provided up to $25 million in total funding for the Commonwealth of Pennsylvania over seven years (2017-2024).^1^ The funding is contingent on the Commonwealth meeting defined participation, financial, and quality targets each performance year.^1^ If the targets are not met, CMMI can reduce funding, take corrective action, or discontinue support. In 2020, CMMI reduced funding as the Commonwealth failed to meet hospital participation scale targets.^3^

The Pennsylvania Department of Health was the agency within the Commonwealth responsible for the administration of PARHM until Act 108 of 2019 established the Pennsylvania Rural Health Redesign Center Authority (RHRCA).^5^ The RHRCA oversees the implementation and methodologies of global budgets, selects hospitals for participation, approves hospital transformation plans, establishes contracts with payers, and provides regulatory oversight.^5^ It also offers technical assistance to participating hospitals, collects and maintains data from participating payers and hospitals, and provides an annual assessment of each participating hospital's compliance with the hospital transformation plan and global budget targets.^5^ Additionally, the RHRCA assesses the progress of participating hospitals in achieving population health goals.^3^

### Components

#### Global Budgets

During each year of PARHM, RHRCA prospectively sets the global budget for each participating rural hospital. This budget is determined primarily based on each hospital's historical net inpatient and outpatient revenue across all participating payers.^1^ Subsequently, each participating payer will remunerate the participating rural hospitals for all inpatient and outpatient hospital-based services, according to the payer's respective share of this global budget. This effectively establishes a revenue agreement with each payer, which aggregates up to the hospital’s annual global budget under PARHM. Pennsylvania's approach to computing the global budgets is subject to review and approval by CMMI.^3^ The global budgets include two components:

1. Fixed Global Budget Payment^1-3^

The fixed payment covers services provided to Medicare fee-for-service patients. Hospitals receive a fixed amount biweekly throughout the year, set in advance by the prospectively set global budget. For PPS hospitals, this amount is pinned to the initial global budget amount. For CAHs, this amount is based on the previous year’s cost report. CAHs with swing-bed approval also receive Medicare reimbursement for post-hospital skilled nursing facility care.

1. Virtual Global Budget Payment^1-3^

The virtual payment covers services provided to patients with coverage from participating commercial payers. While the global budget is still set prospectively, commercial payers elected to make three types of payments to participating hospitals: 1) an upfront float payment equivalent to one month's global budget at the beginning of the year; 2) continued fee-for-service payments for services rendered; 3) if the total payments do not keep pace with the projected global budget, additional payments are provided to ensure that hospitals receive the full global budget. At the end of the year, payers conduct a settlement to the prospective global budget following six months of claims run out to account for any market shifts that may have occurred during the year.

Following each year, there is a reconciliation process.^3^ CAH payments are reconciled to cost-based reimbursement in the case of any over- or under-payments during the year. PPS hospital payments are adjusted based on several factors, including unplanned volume shifts, payer mix shifts, planned changes to service lines, and other adjustments (e.g., COVID-19 Provider Relief Fund Payments, quality targets, etc.). The reconciliation process also includes adjustments for any planned service line changes or unplanned volume shifts.^1-3^

Included and Excluded Hospital Services in the Global Budget^1-3^

Included Services

- Inpatient hospital services
- Outpatient hospital services
  - Emergency department
  - Laboratory
  - Imaging
  - Evaluation and management services
  - Same day surgery
  - Ambulance
  - Other outpatient services
- Critical Access Hospital swing bed services

Excluded Services

- Professional services (inpatient and outpatient)
- Clinic services, including those provided by rural health clinics, community mental health clinics, and federally qualified health centers
- Swing bed services at rural Prospective Payment System hospitals
- Dental services
- Durable medical equipment
- Home health services
- Services provided in dialysis facilities, Indian Health Service facilities, skilled nursing facilities, ambulatory surgery centers, and other specialty facilities

According to the PARHM Annual Reports, “Inpatient professional services are physician services furnished during an inpatient stay. Outpatient professional services are physician services furnished in an outpatient setting (e.g., hospital-based outpatient department). Swing beds are hospital beds that can be used to provide either acute hospital care or post-hospital skilled nursing facility care.”^1-3^

#### Hospital Transformation Plans

Enrolling in PARHM requires that participating hospitals formulate and gain approval for a strategy of how they will reconfigure care delivery. These plans for hospital transformation focus on preventive care and services tailored to the specific needs of the local population, placing a strong emphasis on investing in population health management. The primary goals include reducing avoidable emergency department visits and acute hospitalizations while enhancing overall population health.^1-3^

Rural hospitals engaged in the program are required to submit annual updates on their transformation plans, detailing completed activities and modifications made during the previous year. The Commonwealth evaluates each hospital's progress against the objectives outlined in the transformation plan and issues an annual evaluation report to each hospital. While hospital-specific transformation plans have not been made publicly available, many have chosen to focus on improved care coordination to reduce potentially avoidable utilization. The number of hospitals focused on specific transformation goals and their activities is listed in the PARHM Third Annual Report.^3^

No upfront funding was provided to help hospitals meet program goals. Specifically for hospital transformation plans, savings from the global budget payments were intended to support these plans. However, reports indicate that funding these plans has been a challenge for participating hospitals and they have had to pull from multiple funding streams to facilitate these activities.^3^ Furthermore, differences in hospitals’ baseline financial performance could have affected the capacity to invest in implementing these changes.

## Potentially Avoidable Utilization Incentives

One of the goals of PARHM was to reduce PAU and to provide and better coordinate services outside the hospital. The model included a shared savings feature that was designed to share cost savings (from reduced PAU) between participating hospitals and payers.^3^ Specifically, in each budget year, a hospital’s budget is reduced by a prespecified rate (typically 25-50% of the previous year’s PAU reduction) that reflects anticipated savings from reductions in PAU, thereby transferring these savings to payers.^6^ A hospital retains savings by lowering PAUs below the benchmark. As PARHM continues, the amount a hospital gets to keep of the savings is lowered, as the cost savings are distributed to payers as well.^6^

PAU rates and benchmarks were calculated and managed separately for inpatient and emergency department service lines, and the lower the PAU rate, the better. Inpatient PAU rates are calculated as the revenue from avoidable hospitalizations (readmissions and AHRQ prevention quality indicators—the same measure used in our manuscript) divided by the total inpatient revenue.^6^ The emergency department PAU rate is calculated as the revenue from avoidable emergency department visits divided by total emergency department revenue.^6^

## Changes in Response to COVID-19

The model made several adjustments to account for the impact of the COVID-19 pandemic on participating hospitals. These included the discontinuation of planned quality measurement requirements, hospital participation scale targets, transformation plan deadlines and reporting, and temporary suspension of global budget reconciliation until data on the scope of the pandemic's impact became available.^2^

More specifically, the All-Payer Quality Program, originally intended for global budget adjustments based on hospital performance, was canceled due to resource shortages during the pandemic; instead, existing Medicare quality programs were used to determine global budget adjustments.^2,3^ Furthermore, the deadline for hospital transformation plans was extended and allowed to exclude supplementary information on plan progress.^2^ The global budget underwent adjustments, incorporating the suspension of sequestration of funding through the CARES Act Provider Relief Fund.^2^ Considerations for adjusting the Medicare FFS portion of global budgets to account for COVID-19 were deferred until a later date.

## PARHM Participants

Five hospitals joined the model in 2019 (Cohort 1), eight in 2020 (Cohort 2), and five in 2021 (Cohort 3) for a total of 18 hospitals.^3^ Participating payers included: Geisinger, Highmark Blue Cross Blue Shield, University of Pittsburgh Medical Center, Aetna, Highmark Wholecare, and Medicare fee-for-service.^3^


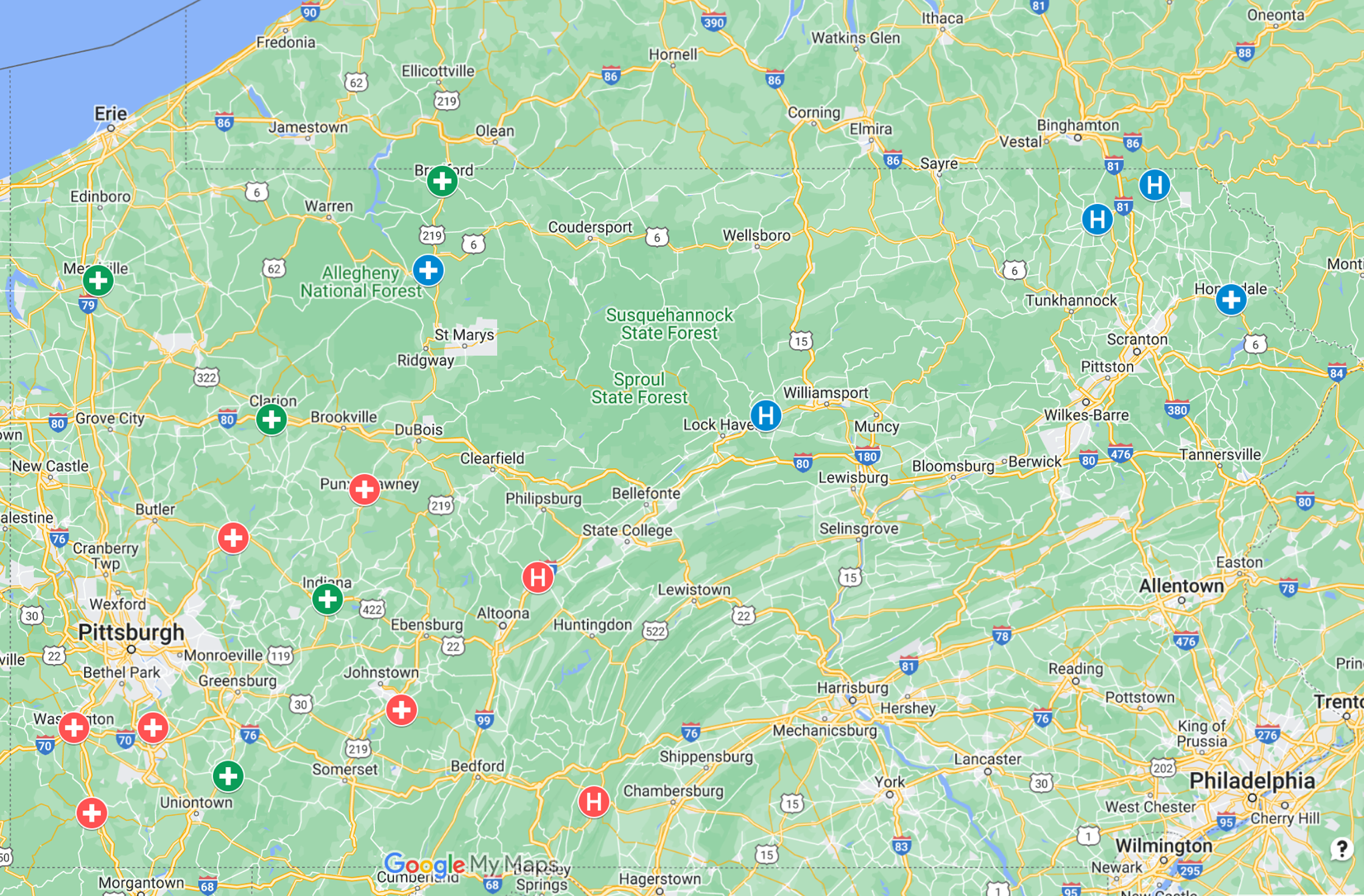


### Appendix 1 Figure 1. Map of Participating Hospitals

Source: Author generated custom Google My Maps

Key: H = Critical Access Hospital; + = Prospective Payment System Hospital; Blue= Cohort 1; Red= Cohort 2; Green = Cohort 3

### Appendix 1 Table 1. List of Participating Hospitals

| **Cohort Joined** | **Hospital Name** | **Hospital Type** |
| --- | --- | --- |
| 1 | Wayne Memorial Hospital | PPS |
| 1 | Jersey Shore Hospital | CAH |
| 1 | UPMC Kane Community Hospital | PPS |
| 1 | Endless Mountains Health Systems | CAH |
| 1 | Barnes-Kasson County Hospital | CAH |
| 2 | Armstrong Center for Medicine & Health Hospital | PPS |
| 2 | Fulton County Medical Center | CAH |
| 2 | Monongahela Valley Hospital | PPS |
| 2 | Punxsutawney Area Hospital | PPS |
| 2 | Tyrone Hospital | CAH |
| 2 | Washington Hospital | PPS |
| 2 | Southwest Regional Medical Center | PPS |
| 2 | Chan Soon-Shiong Medical Center at Windber | PPS |
| 3 | Bradford Regional Medical Center | PPS |
| 3 | Clarion Hospital | PPS |
| 3 | Highlands Hospital | PPS |
| 3 | Indiana Regional Medical Center | PPS |
| 3 | Meadville Medical Center | PPS |

Source: CMS PARHM Fact Sheet

Abbreviations: CAH = Critical Access Hospital; PPS = Prospective Payment System Hospital

### Participation Goals

The model aimed to achieve participation targets (termed revenue scale targets) over time. The targets were for global budgets to cover at least 75 percent of each participating hospital’s eligible net revenue in 2019 and at least 90 percent of each participating hospital’s eligible net revenue each year between 2020 and 2024.^1^

In 2019, three out of the five participating hospitals meet their revenue scale targets.^1^ In response to the COVID-19 pandemic beginning in 2020, CMMI approved a request to delay the reconciliation of the 2020 global budget and complete it at the same time as the 2021 budget.^3^ However, these results have yet to be reported at the time of writing.

# Appendix 2: Detailed description of data sources

American Community Survey (ACS)

ACS data was be used to capture area-level measures of socioeconomic status, including education, household income, and poverty, measured at the ZIP Code Tabulation Area level to control for heterogeneity in patient characteristics that vary across geographic areas and that are associated with patients’ use of hospital care. This data was then coded up to the HSA-level.

The Dartmouth Atlas of Health Care (DA)

The Dartmouth Atlas uses Medicare and Medicaid data to provide information and analysis about national, regional, and local markets, as well as hospitals. Hospital service areas (HSAs) are local healthcare markets for hospital care. An HSA is a collection of ZIP codes whose residents receive most of their hospitalizations from the hospitals in that area. HSAs were defined by assigning ZIP codes to the hospital area where the greatest proportion of their Medicare residents were hospitalized. We assigned each hospital to their corresponding HSA number based on 2019 HSA codes (the last available numbers) across all years to maintain stable geographic boundaries.

CMS COVID-19 Data

The COVID-19 Reported Patient Impact and Hospital Capacity by Facility dataset provides facility-level data for hospital utilization aggregated on a weekly basis. These are derived from reports with facility-level granularity across two main sources: (1) Health and Human Services (HHS) TeleTracking, and (2) reporting provided directly to HHS Protect by state/territorial health departments on behalf of their healthcare facilities. The hospital population includes all hospitals registered with Centers for Medicare & Medicaid Services (CMS) as of June 1, 2020. We assigned each hospital to their corresponding HSA number and aggregated data up to the HSA level for analyses.

Pennsylvania Health Care Cost Containment Council Database (PHC4)

PHC4 is an independent Pennsylvania state agency that collects data on healthcare in the state. All licensed healthcare facilities are required to report administrative data to PHC4 on a quarterly basis and data are validated by standard processes and verified by the reporting facility. These data include information on Diagnosis Related Groups, Major Diagnostic Categories, admission type and source, diagnosis and procedure codes, discharge status, length of stay, payer, facility, and financial characteristics. This study includes PHC4 data from 2016 quarter 1 through 2022 quarter 4. PHC4 data was used to construct PAU rates (i.e., Prevention Quality Indicators: Overall, Acute, Chronic, and Diabetes Composite measures).

# Appendix 3: Agency for Healthcare Research and Quality Prevention Quality Indicators

## Appendix 3 Table 1. Measures of Potentially Avoidable Utilization

| **Potentially avoidable hospitalizations (overall measure), measured using the AHRQ Prevention Quality Indicators Overall Composite Measure (PQI 90)** |
| --- |
| PQI 01 Diabetes Short-Term Complications Admission Rate |
| PQI 03 Diabetes Long-Term Complications Admission Rate |
| PQI 05 Chronic Obstructive Pulmonary Disease (COPD) or Asthma in Older Adults Admission Rate |
| PQI 07 Hypertension Admission Rate |
| PQI 08 Heart Failure Admission Rate |
| PQI 11 Community Acquired Pneumonia Admission Rate |
| PQI 12 Urinary Tract Infection Admission Rate |
| PQI 14 Uncontrolled Diabetes Admission Rate |
| PQI 15 Asthma in Younger Adults Admission Rate |
| PQI 16 Lower-Extremity Amputation among Patients with Diabetes Rate |
| **Potentially avoidable hospitalizations (acute measure), measured using the AHRQ Prevention Quality Indicators Acute Composite Measure (PQI 91)** |
| PQI 11 Bacterial Pneumonia Admission Rate |
| PQI 12 Urinary Tract Infection Admission Rate |
| **Potentially avoidable hospitalizations (chronic measure), measured using the AHRQ Prevention Quality Indicators Chronic Composite Measure (PQI 92)** |
| PQI 01 Diabetes Short-Term Complications Admission Rate |
| PQI 03 Diabetes Long-Term Complications Admission Rate |
| PQI 05 Chronic Obstructive Pulmonary Disease (COPD) or Asthma in Older Adults Admission Rate |
| PQI 07 Hypertension Admission Rate |
| PQI 08 Heart Failure Admission Rate |
| PQI 14 Uncontrolled Diabetes Admission Rate |
| PQI 15 Asthma in Younger Adults Admission Rate |
| PQI 16 Lower-Extremity Amputation among Patients with Diabetes Rate |
| **Potentially avoidable hospitalizations (diabetes measure), measured using the AHRQ Prevention Quality Indicators Diabetes Composite Measure (PQI 93)** |
| PQI 01 Diabetes Short-Term Complications Admission Rate |
| PQI 03 Diabetes Long-Term Complications Admission Rate |
| PQI 14 Uncontrolled Diabetes Admission Rate |
| PQI 16 Lower-Extremity Amputation among Patients with Diabetes Rate |

Source: Agency for Healthcare Research and Quality Prevention Quality Indicators version 2022

Notes: For technical specifications of each PQI and instructions on how to calculate composite measures, see Agency for Healthcare Research and Quality’s Quality Indicator User Guide.^7^

Abbreviations: PQI= Prevention Quality Indicator

# Appendix 4: Detailed overview of our statistical approach

Propensity score weighting can be used to reduce the effects of observed confounding when using observational data to estimate the effects of exposures, like PARHM.^8^ To generate our propensity score weights, we ran a logistic regression to predict PARHM participation as a function of HSA level demographics and hospital characteristics. We tested for multicollinearity and removed covariates with a variance inflation factor greater than five.^9^ This process was repeated until all included covariates had a variance inflation factor no greater than 5 (the variance inflation factor reflects the extent to which a given covariate can be expressed as a linear combination of others—i.e., multicollinearity—with higher variance inflation factors reflecting greater multicollinearity). Based on the included variables (Appendix 4 Table 1) we calculated propensity scores in the pre-PARHM period (2016-2018) and carried forward through the post-PARHM period.

Propensity scores were then incorporated into the generation of the average treatment effect on the treated (ATT) weights. We used this approach to weight the comparison group to resemble the treated group on baseline covariates. Propensity score weighting helps to adjust for observed baseline differences between HSAs whose hospitals did vs. did not participate in PARHM, which can introduce bias in DiD designs if these baseline differences were correlated with outcome trends. Under the assumptions of the DiD design, differential changes in outcomes between the treatment and groups can then be attributed to the introduction of global budgets under PARHM.^10^

The Callaway and Sant’anna DiD approach aggregates DiD estimates over sub-experiments, or “stacks,” each of which contains a cohort of treated HSAs that entered PARHM in a particular year compared to control HSAs (i.e., HSAs with never-treated hospitals). Estimates are pooled across stacks to yield an overall DiD estimate. We estimated a linear model, with the HSA-year as the unit of analysis, of the form:

$$Y_{its}=\beta_{0}+\delta\left( {Post}_{its}* {PARHM}_{is} \right)+\beta_{1}X_{its}\left( {Pre}_{its} \right)+ \mu_{is}+ \tau_{ts}+ \varepsilon_{its} (1)$$

As indicated in Equation 1, $Y_{its}$ is the PAU rate in HSA $i$ in year $t$ in stack $s$; ${Post}_{its}$ indicates the post PARHM period in HSA $i$ in year $t$ in stack $s$; ${PARHM}_{is}$ indicates if a hospital in that HSA participated in PARHM during the study period; and $X_{ist}$ is a vector including controls for time-varying HSA covariates (e.g., HSA-level demographics and hospital characteristics) in the pre-intervention period $\left( {Pre}_{its} \right)$. HSA fixed effects ($\mu_{is}$) control for constant differences across HSAs (e.g., differences in hospital size and finances) and secular trends ($\tau_{ts}$). The parameter of interest $\delta$ is an overall DiD estimate representing an average effect of PARHM, pooled across all HSAs with participating hospitals. All statistical analyses were performed with Stata 18.0 (StataCorp, College Station, TX). A P-value of less than 0.05 was considered significant.

## Appendix 4 Table 1. Standardized Mean Differences of Covariates with Average Treatment Effect on the Treated and Propensity Score Weighting

| **Hospital Service Area Characteristics** | **Before Weighting** | **After Weighting** |
| --- | --- | --- |
| Total Population, (thousands) | -0.467* | -0.076 |
| Median Income, ($ thousands) | 0.040 | -0.010 |
| High School Grad, % | 0.216* | -0.092 |
| Unemployed, % | 0.130 | 0.052 |
| Non-white, % | 0.022 | -0.180 |
| Uninsured, % | -0.082 | 0.489* |
| Below Poverty, % | -0.130 | 0.214* |
| Married, % | -0.026 | 0.210* |
| ADI^†^ | 0.173 | 0.125 |
| CARR^†^ | 0.256* | 0.115 |
| FORHP Status | 0.628* | 0.185 |
| Critical Access Hospital, n (%) | 0.186 | 0.247* |
| Independent Hospital^‡^, n (%) | 1.100* | 0.365* |
| 340B Participant, n (%) | 0.354* | -0.205* |
| Inpatient Discharge Rate, (per 100k) | -0.225* | 0.064 |
| Outpatient Discharge Rate, (per 100k) | -0.191 | -0.029 |
| Total Margin^§^, % | -0.001 | 0.001 |
| Uncompensated Care^‖^, % | <0.001 | <0.001 |
| Medicare share of NPR^¶^, % | <0.001 | <0.001 |
| Medicaid Share of NPR^#^, % | <0.001 | <0.001 |

*Values ≥ 0.2 in magnitude are considered statistically significant

^†^ z-scored

^‡^ Independent (i.e., not merged or affiliated with another hospital or system)

^§^ The ratio of total income to total revenue
^‖^ Percent of uncompensated care (charity care and bad debt) relative to net patient revenue

^¶^ Percent of Medicare revenue relative to net patient revenue

^#^ Percent of Medicaid revenue relative to net patient revenue

Source: Authors’ analysis of American Community Survey and Pennsylvania Health Care Cost Containment Council data (2016-2018)

Abbreviations: PARHM= Pennsylvania Rural Health Model; NPR= Net Patient Revenue; ADI= Area Deprivation Index; CARR= Community Assets and Relative Rurality Index; FORHP= Federal Office of Rural Health Policy

## Appendix 4 Figure 1. Distribution of Propensity Scores

Source: Authors’ analysis of American Community Survey and Pennsylvania Health Care Cost Containment Council data (2016-2018)

Abbreviations: PARHM= Pennsylvania Rural Health Model; HSA= Hospital Service Area; ATT= Average Treatment Effect on the Treated

# Appendix 5: Baseline Period Data

## Appendix 5 Table 1. Pre- and Post-Treatment Potentially Avoidable Hospitalization Rates per 100,000 people per Hospital Service Area-Year

| **Potentially Avoidable**  **Hospitalizations** | **Pre-Treatment Average** | **Post-Treatment Average** | **Difference** | **Difference in Differences** |
| --- | --- | --- | --- | --- |
| **Overall Composite** |  |  |  |  |
| Treated HSAs | 584.07 | 386.36 | -197.70 | -20.26 |
| Control HSAs | 582.36 | 404.91 | -177.45 |  |
| P-value | 0.9551 | 0.4375 |  |  |
| **Acute Composite** |  |  |  |  |
| Treated HSAs | 146.84 | 106.52 | -40.32 | -12.08 |
| Control HSAs | 98.89 | 70.65 | -28.24 |  |
| P-value | <0.0001 | <0.0001 |  |  |
| **Chronic Composite** |  |  |  |  |
| Treated HSAs | 437.22 | 279.84 | -157.38 | -8.18 |
| Control HSAs | 483.46 | 334.26 | -149.20 |  |
| P-value | 0.0707 | 0.0066 |  |  |
| **Diabetes Composite** |  |  |  |  |
| Treated HSAs | 37.05 | 40.19 | 3.14 | -4.39 |
| Control HSAs | 52.52 | 60.05 | 7.53 |  |
| P-value | <0.0001 | <0.0001 |  |  |

Source: Authors’ analysis of Pennsylvania Health Care Cost Containment Council data (2016-2022)

Notes: Results are population-weighted.

Abbreviations: HSA= Hospital Service Area

# Appendix 6: Secondary Analyses

## Appendix 6 Figure 1. Event Study Graph for Prevention Quality Indicator Acute Composite Rates per 100,000 people per Hospital Service Area-Year

Source: Authors’ analysis of Pennsylvania Health Care Cost Containment Council data (2016-2022)

Notes: Point estimates are displayed along with their 95% confidence intervals. Year 0 represents the first year of PARHM enrollment (i.e., 2019 for Cohort 1 HSAs, 2020 for Cohort 2 HSAs, 2021 for Cohort 3 HSAs). Reference periods are the year before PARHM enrollment, set to zero, and omitted from the graph.

Abbreviations: PARHM= Pennsylvania Rural Health Model; PQI= Prevention Quality Indicator.

## Appendix 6 Figure 2. Event Study Graph for Prevention Quality Indicator Chronic Composite Rates per 100,000 people per Hospital Service Area-Year

Source: Authors’ analysis of Pennsylvania Health Care Cost Containment Council data (2016-2022)

Notes: Point estimates are displayed along with their 95% confidence intervals. Year 0 represents the first year of PARHM enrollment (i.e., 2019 for Cohort 1 HSAs, 2020 for Cohort 2 HSAs, 2021 for Cohort 3 HSAs). Reference periods are the year before PARHM enrollment, set to zero, and omitted from the graph.

Abbreviations: PARHM= Pennsylvania Rural Health Model; PQI= Prevention Quality Indicator.

## Appendix 6 Figure 3. Event Study Graph for Prevention Quality Indicator Diabetes Composite Rates per 100,000 people per Hospital Service Area-Year

Source: Authors’ analysis of Pennsylvania Health Care Cost Containment Council data (2016-2022)

Notes: Point estimates are displayed along with their 95% confidence intervals. Year 0 represents the first year of PARHM enrollment (i.e., 2019 for Cohort 1 HSAs, 2020 for Cohort 2 HSAs, 2021 for Cohort 3 HSAs). Reference periods are the year before PARHM enrollment, set to zero, and omitted from the graph.

Abbreviations: PARHM= Pennsylvania Rural Health Model; PQI= Prevention Quality Indicator.

# Appendix 7: Sensitivity and Subgroup Analyses

## Appendix 7 Table 1. Sensitivity Analyses: Difference-in-Differences Estimates (Potentially Avoidable Hospitalization Rates per 100,000 people per Hospital Service Area-Year) Pooled and Across Individual Cohorts

| **Overall Composite** | **PARHM Cohort 1**  ATT (95% CI) | **PARHM Cohort 2**  ATT (95% CI) | **PARHM Cohort 3**  ATT (95% CI) | **Pooled**  ATT (95% CI) |
| --- | --- | --- | --- | --- |
| Treatment Anticipation:  Alternate Start Date | -5.32  (-142.16 to 131.53) | -46.05  (-118.71 to 26.61) | 15.81  (-60.05 to 91.67) | -19.75  (-88.11 to 48.61) |
| COVID-19:  Removal of 2020-2021 data | -18.23  (-88.31 to 51.84) | -49.35  (-111.37 to 12.66) | 56.95  (-19.00 to 132.89) | -7.04  (-56.04 to 41.95) |
| Alternative Estimation Method: OLS | -27.45  (-138.12 to 83.22) | -67.50*  (-103.32 to -31.67) | 47.03*  (16.70 to 77.37) | -28.36  (-68.82 to 12.11) |
| Alternative Estimation Method: IPW | -27.97  (-145.10 to 89.16) | -70.84*  (-113.62 to -28.07) | 46.21  (15.86 to 76.57) | -30.41  (-72.48 to 11.69) |
| Alternative Estimation Method: IPW with stabilized weights | -27.82  (-142.40 to 86.76) | -66.42*  (-99.40 to -33.43) | 46.10*  (15.79 to 76.42) | -28.13  (-67.94 to 11.69) |
| Alternative Estimation Method: Not Yet Treated as Controls | -27.14  (-122.56 to 68.27) | -63.62*  (-123.69 to -3.55) | 46.99  (-11.57 to 105.55) | -26.30  (-64.62 to 12.02) |
| Partially Unadjusted: No Covariates | -28.75  (-155.92 to 98.41) | -70.86  (-159.34 to 17.63) | 50.83  (-10.53 to 112.19) | -29.37  (-80.73 to 22.00) |
| Fully Unadjusted: No covariates or ATT/ propensity score weighting | -21.07  (-137.15 to 95.01) | -40.39  (-82.41 to 1.63) | 18.57  (-11.75 to 48.89) | -20.52  (-58.26 to 17.22) |

*Significant at the *P* < 0.05 level

Source: Authors’ analysis of Pennsylvania Health Care Cost Containment Council data (2016-2022)

Notes: ‘Pooled’ reports the aggregated measure of treatment effects across all cohorts; Alternate start date involved moving the start date up by 1-year; COVID-19 involved removing data from the year 2020-2021; ‘Not Yet Treated as Controls’ includes the never treated as well as cohorts that have not been treated yet, but will eventually become treated (e.g., Cohort 2 and 3 HSAs were used as controls for Cohort 1 HSAs until they enrolled in PARHM).

Abbreviations: PARHM= Pennsylvania Rural Health Model; ATT= Average Treatment Effect on the Treated; PQI= Prevention Quality Indicator; OLS= ordinary least squares; IPW= Inverse probability weighting.

## Appendix 7 Table 2. Subgroup Analyses: Difference-in-Differences Estimates (Potentially Avoidable Hospitalization Rates per 100,000 people per Hospital Service Area-Year) Pooled and Across Individual Cohorts

| **Overall Composite** | **PARHM Cohort 1**  ATT (95% CI) | **PARHM Cohort 2**  ATT (95% CI) | **PARHM Cohort 3**  ATT (95% CI) | **Pooled**  ATT (95% CI) |
| --- | --- | --- | --- | --- |
| FORHP Rural | -11.96  (-158.62 to 134.69) | -94.28  (-196.52 to 7.95) | 53.40  (-23.55 to -130.35) | -20.78  (-96.02 to 54.45) |
| PPS | 58.09  (-34.13 to 150.31) | -74.69*  (-137.19 to -12.20) | 45.91  (-20.76 to 112.59) | -17.23  (-56.49 to 22.02) |
| CAH | -138.27*  (-249.22 to -27.32) | 239.46  (-167.61 to 646.52) | N/A | 7.53  (-216.94 to 232.01) |

*Significant at the *P* < 0.05 level

Source: Authors’ analysis of Pennsylvania Health Care Cost Containment Council data (2016-2022)

Notes: ‘Pooled’ reports the aggregated measure of treatment effects across all cohorts. FORHP’s definition includes all non-metro counties, all metro census tracts with Rural-Urban Commuting Area (RUCA) codes 4-10, and large area metro census tracts of at least 400 square miles in areas with populations density of 35 or less per square mile with RUCA codes 2-3. In the FORHP analysis there were four Cohort 1 HSAs, six Cohort 2 HSAs, four Cohort 3 HSAs, and 21 control HSAs. In the PPS analysis there were two Cohort 1 HSAs, six Cohort 2 HSAs, five Cohort 3 HSA, and 35 control HSAs. In the CAH analysis there were three Cohort 1 HSAs, two Cohort 2 HSAs, zero Cohort 3 HSA, and 10 control HSAs.

Abbreviations: PARHM= Pennsylvania Rural Health Model; ATT= Average Treatment Effect on the Treated; FORHP= Federal Office of Rural Health Policy; PPS= Health Service Areas with a Prospective Payment System hospital; CAH= Health Service Areas with a Critical Access Hospital; N/A= Not Applicable

# Appendix 8: COVID-19 Data

## Appendix 8 Table 1. Total Confirmed COVID-19 Admissions

| **Year** | **Control HSAs** | **Treated**  **HSAs** | **PARHM Cohort 1** | **PARHM Cohort 2** | **PARHM Cohort 3** |
| --- | --- | --- | --- | --- | --- |
| 2016 | 0 | 0 | 0 | 0 | 0 |
| 2017 | 0 | 0 | 0 | 0 | 0 |
| 2018 | 0 | 0 | 0 | 0 | 0 |
| 2019 | 0 | 0 | 0 | 0 | 0 |
| 2020 | 9506 | 2677 | 219 | 1464 | 994 |
| 2021 | 20491 | 6873 | 844 | 3347 | 2682 |
| 2022 | 11537 | 4060 | 448 | 1579 | 2033 |

Source: Authors’ analysis of the COVID-19 Reported Patient Impact and Hospital Capacity by Facility dataset (2020-2022)

Abbreviations: PARHM= Pennsylvania Rural Health Model; HSA= Hospital Service Area

## Appendix 8 Table 2. Confirmed COVID-19 Admission Rate per 100,000 people per Hospital Service Area-Year

| **Year** | **Control HSAs** | **Treated**  **HSAs** | **T-test** | **PARHM Cohort 1** | **PARHM Cohort 2** | **PARHM Cohort 3** | **ANOVA** |
| --- | --- | --- | --- | --- | --- | --- | --- |
| 2016 | 0 | 0 | - | 0 | 0 | 0 | - |
| 2017 | 0 | 0 | - | 0 | 0 | 0 | - |
| 2018 | 0 | 0 | - | 0 | 0 | 0 | - |
| 2019 | 0 | 0 | - | 0 | 0 | 0 | - |
| 2020 | 436.10 | 478.89 | 0.812 | 272.30 | 581.02 | 522.06 | 0.082 |
| 2021 | 856.30 | 1258.86 | 0.201 | 900.43 | 1337.55 | 1491.38 | 0.474 |
| 2022 | 449.74 | 658.76 | 0.308 | 441.64 | 441.64 | 1192.32 | 0.176 |

Source: Authors’ analysis of the COVID-19 Reported Patient Impact and Hospital Capacity by Facility dataset (2020-2022)

Abbreviations: PARHM= Pennsylvania Rural Health Model; HSA= Hospital Service Area; ANOVA= Analysis of Variance

# References

1. NORC at the University of Chicago. *The Pennsylvania Rural Health Model (PARHM) First Annual Report.* 2021.

2. NORC at the University of Chicago. *The Pennsylvania Rural Health Model (PARHM) Second Annual Report.* 2022.

3. NORC at the University of Chicago. *The Pennsylvania Rural Health Model (PARHM) Third Annual Report.* 2023.

4. Services; CfMM. Pennsylvania Rural Health Model. <https://www.cms.gov/priorities/innovation/innovation-models/pa-rural-health-model>. Published 2023. Accessed December 1, 2023.

5. Center; RHR. *Rural Legislative Summer Webinar: Transforming Rural Health Care in Pennsylvania through Innovation in Payment and Service.*  August 17, 2022 2022.

6. Rural Health Redesign Center: All Provider Call. In:2021.

7. American Hospital Association. Rural report. <https://www.aha.org/system/files/2019-02/rural-report-2019.pdf>. Published 2019. Accessed July 1, 2023.

8. Austin PC. Variance estimation when using inverse probability of treatment weighting (IPTW) with survival analysis. *Stat Med.* 2016;35(30):5642-5655.

9. Kim JH. Multicollinearity and misleading statistical results. *Korean J Anesthesiol.* 2019;72(6):558-569.

10. Li X, Shen C. Doubly robust estimation of causal effect: upping the odds of getting the right answers. *Circulation: Cardiovascular Quality and Outcomes.* 2020;13(1):e006065.
